# The Link Between Rheumatoid Arthritis and Dementia: A Review

**DOI:** 10.7759/cureus.7855

**Published:** 2020-04-27

**Authors:** Pritpal S Sangha, Mala Thakur, Zaiba Akhtar, Shaun Ramani, Rubby S Gyamfi

**Affiliations:** 1 Medicine, Medical University of Lublin, Lublin, POL; 2 Internal Medicine, Xavier University School of Medicine, Oranjestad, ABW; 3 Internal Medicine, American University School of Medicine Aruba, Oranjestad, ABW; 4 Internal Medicine, American University of Integrative Sciences School of Medicine, St. Michael, BRB

**Keywords:** alzheimer's disease, dementia, rheumatoid arthritis, autoimmune disease

## Abstract

Background: This study explored the relationship between rheumatoid arthritis (RA) and dementia. These two diseases are a significant health burden that affects the older population, although they have also manifested in young people to a smaller extent.

Methodology: The study entailed a detailed literature review of articles on RA and dementia. The peer-reviewed articles were sourced from reputable databases such as Research Gate, National Center for Biotechnology Information, PubMed, and Google Scholar.

Results: RA is a chronic disorder that affects millions of Americans. Dementia, on the other hand, is associated with diminishing cognitive capabilities that impair daily living. Both diseases are associated with older persons and genetic factors. Besides, the inflammation associated with RA reduced blood flow to vital body organs, which increases the risk of developing dementia. Additionally, the study revealed that medications used by RA patients increase the risk of developing dementia. However, biological therapies such as tumor necrosis factor (TNF) inhibitors can lower the risk of dementia.

Conclusion: There is a need to develop diagnostic procedures that will enable early diagnosis and commencement of treatment to slow down the progression of both disorders. Furthermore, managing these disorders effectively mandates increased awareness about the causality and risk factors of both diseases, especially among young people and at-risk populations to promote lifestyle change and increased uptake of primary care services.

## Introduction and background

Rheumatoid arthritis (RA) is a chronic disorder that is characterized by the body's immune system releasing antibodies that attack body tissues and organs thereby resulting in painful inflammation. The illness is one of the most common chronic systemic inflammatory disorders that affects the joints and is characterized by the inflammation of the synovial membrane. It also manifests in the hand, feet, and cervical spine and other vital organs such as the heart and lungs (15%-25% of diagnosed cases). Studies show that it affects 5-50 per 100000 persons per year [1]. For example, the U.S. has about 1.5 million RA victims [1]. RA is caused by a combination of factors that triggers the body's immune system to release antibodies such as the rheumatoid factor (RF) and anti-cyclic citrullinated peptide antibody that attack joint linings.

On the other hand, dementia is a collective name given to diseases and conditions that result in the deterioration of one's memory, language, problem-solving capacity, thinking skills, and ability to function normally. Although Alzheimer's disease (AD) is the most common form of dementia accounting for 50% of cases; other forms of dementia include Lewy body dementia, frontotemporal dementia, human immunodeficiency virus (HIV), Creutzfeldt-Jakob disease (CJD), syphilis, and normal pressure hydrocephalus. In 2015, there were about 14.47 million dementia patients with an anticipated annual case increment of 7.7 million people [2]. Statistically, this translates to a new case every 4.1 s. Beta-amyloid deposits and intracellular neurofibrillary tangles characterize AD. The former is associated with events such as the loss of neuronal synapses, progressive neurotransmitter deficits, and death of neuronal cells. Neurofibrillary tangles, on the other hand, result in the development of insoluble twisted fibers in the brain cells. The fibers, which are protein cells (tau), form a microtubule that is tasked with transporting nutrients to different parts of the nerve cell. In the case of AD, the tau protein is tampered with, which results in nonfunctional microtubules. The accumulation of the tau proteins in the neurons is promoted by an inflammation mechanism and condition called RA. As such, RA is a risk factor for AD. Thus, the objective of this study is to review and analyze literature on the association between RA and dementia. 

## Review

Dementia

Dementia is a common name for neurological diseases that gradually and permanently impair one's capacity to think and remember things [1]. There is a misconception that dementia affects only old people because of its commonality in geriatric patients. Evidence shows that it also affects young people with juveniles showing significant manifestations [1]. Besides, dementia is considered a syndrome because it alters many body organs that correlate, such as the brain, respiratory system, endocrine system, and muscles and bones, among other vital organs. The alteration of the functions of these organs results in a series of complications such as functional changes of the brain that affect the autonomous functioning of the patient. The treatment of these complications often calls for a multidisciplinary focus. On the other hand, Dr. Barkhof refers to dementia as a clinical syndrome rather than a disease. The doctor explains that it is an acquired condition that is characterized by multiple cognitive impairments that limit the activities of daily living [2]. As such, these explanations illustrate that dementia cannot be considered a common brain disease, but a group of diseases that cause functional alterations of the body's vital organs, which impairs a patient’s normal functioning (social and psychological functioning).

**Figure 1:**
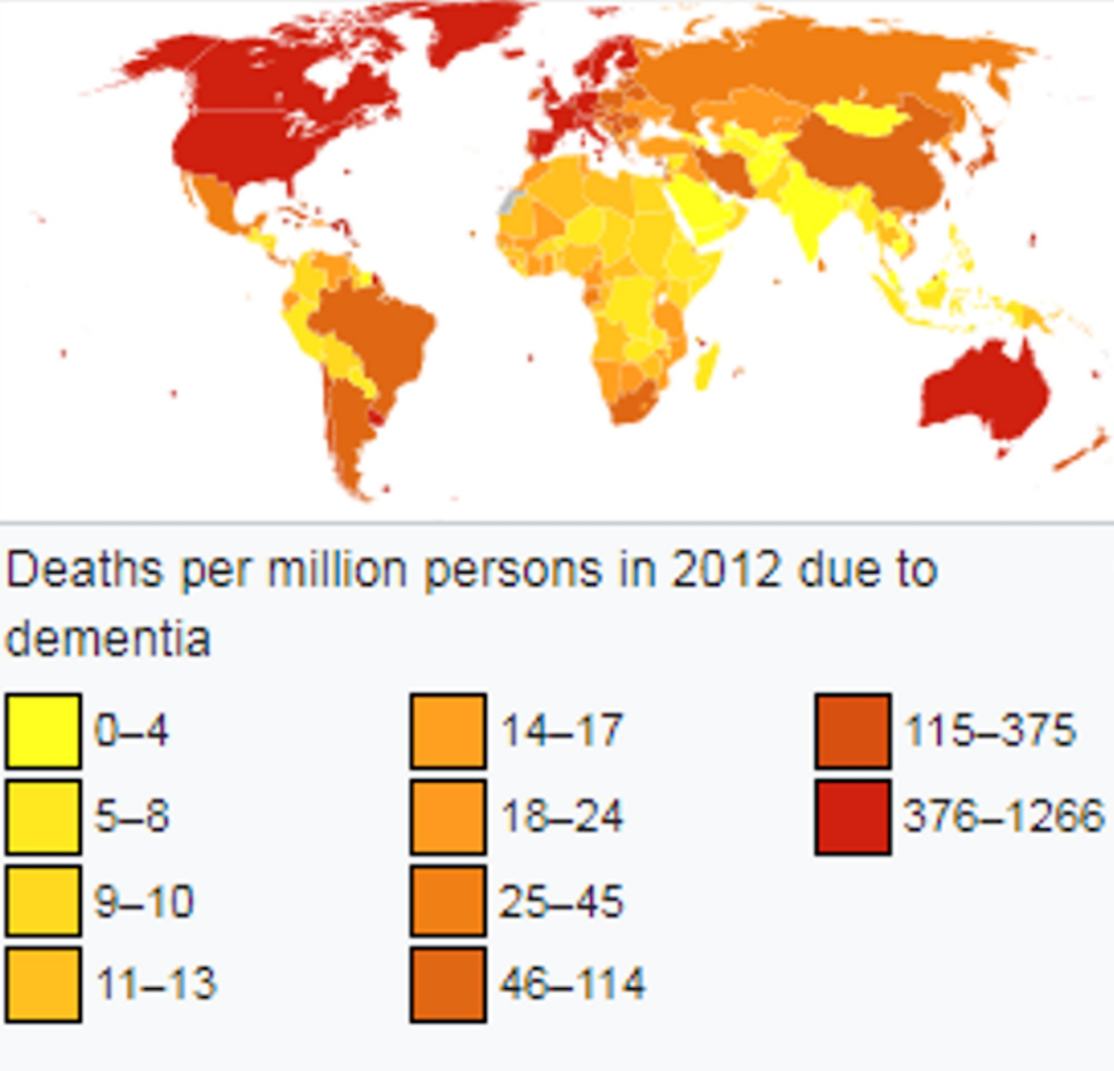
Deaths per million persons in 2012 due to dementia. Courtesy: Extracted from Ref. [2].

Dr. Barkhof reiterated that dementia impairs one's memory, language processing, visual perception, and other executive body functions [2]. The alterations of these body processes make one dependent on his or her principal caretaker. Unfortunately, the onset of dementia cannot be disrupted, but Dr. Barkhof asserts that it is not necessarily progressive. As such, patients should not lose hope once diagnosed with early-stage dementia. Displayed in Figure 1 shows how millions were deadly impacted in 2012 by dementia worldwide. 

As for the causes, Dr. Winbald explains that dementia is age-related; aging increases the probability of developing dementia. The increase in life expectancy and a decrease in the birth rate have resulted in the burgeoning of the older population and a significant increase in dementia cases. Dr. Olney cited a WHO study that revealed that dementia in the older population manifests around the age 65-70. However, some patients show symptoms 10 years earlier before the diagnosis is made [3]. Unfortunately, the assumption that such symptoms are synonymous with growing old makes people delay seeking medical attention until the point when the disease is at an advanced stage. Thus, the delay limits the effective management of dementia. 

Dementia's physiopathology

Many diseases are classified as dementia, including Lewy body dementia and Alzheimer's, among others. Besides, few studies have been conducted to understand these diseases, which has resulted in shallow understanding of their causes, effects, and characteristics. According to a study by Hinz, dementia is caused by misfolded proteins that transmit their shape to healthy proteins, thereby resulting in changes and leads to fatal and genetically transmissible neurodegenerative diseases [4]. The study also revealed that some of the casual factors for dementia are inflammatory mediators' productions that manifest in other conditions such as sarcoidosis, Behcet syndrome, multiple sclerosis, Sjôgren syndrome, among others [5]. The progression of these diseases often affects the functioning of the brain in some way, which is a risk factor for the development of dementia. Other genetic factors included in the study to understand the progression of Alzheimer include amyloid precursor protein (APP), presenilin-1 (PSEN1), and presenilin-2 (PSEN2) [5]. 

**Figure 2:**
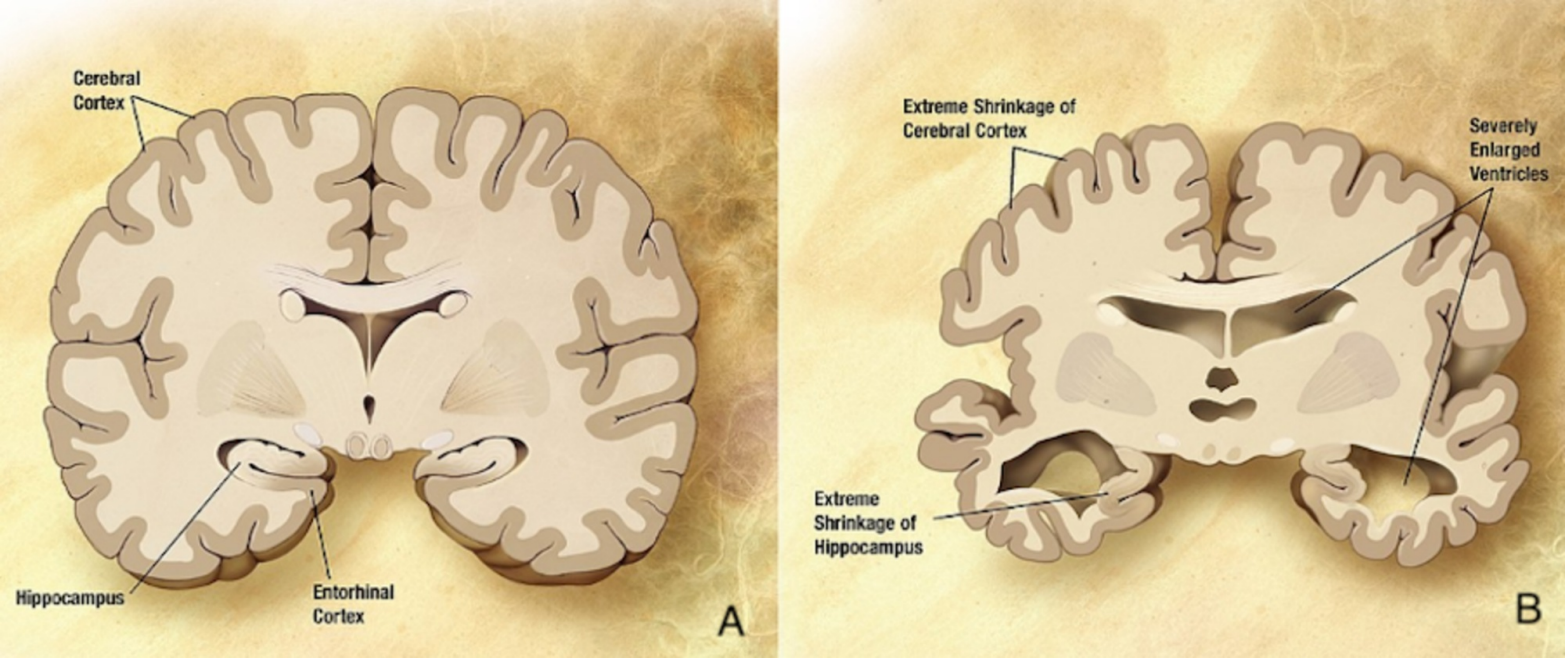
Differences between healthy brain structure (A) and dementia patient's brain structural changes (B). Courtesy: Extracted from Ref. [2].

Dementia is a disease that may have typical or atypical presentation [6]. The most typical course of this disease includes neurological alterations primarily that may lead to other alterations in many areas of the patient’s health. Most of the typical symptoms have something to do with neurological damage, such as tremors, memory loss, and peripheral manifestations as gastrointestinal habit changes. Also, psychiatric manifestations are common, and they appear usually as early symptoms because of the mental impact that this disease has on the patient [6]. According to this author, the principal mental problems that patients suffer, include acute appearance of depression or the exacerbation of an episode of depression, generalized anxiety, suicidal thoughts, to mention the most common ones. In the book “Challenging behavior in dementia: a person-centered approach”, published by Stokes in 2017, it is mentioned that the principal areas of treatment, with a person centered focus, may lead to the importance of psychological therapy, as well as medication, to improve the patient’s quality of life, as well as the correct psychological assessment of the patient’s family, or at least, the principal caretaker. Stokes makes emphasis on the importance of psychological therapy even when the patients are on advanced phases of the disease, because of its proved good results on the improvement of the patient’s quality of life. It is imperative to co-work with specialists in many areas, in order to improve many of the patient’s areas and decrease the accelerated usual curse of this disease. The multidisciplinary treatment is mandatory, as told by Stokes, so the patients get the best and most complete intervention, to slow down a bit the evolution of the dementia, to avoid complications and comorbidities that usually coexist with this disease, and also allow the patient to have a better control of his/her body, until the dementia causes structural or great decrease of brain normal functioning. It is well known that dementia is a chronic, unstoppable disease, so it is impossible to avoid the natural curse of it, but that does not mean that patients have to suffer each phase of the disease. According to Stokes, psychological plus medical treatment may create a more comfortable ambience for the patient and his/her family, in order to provide appropriate closure and better quality of life for everyone. Dementia is a disease that causes many several changes in the patient’s life and, obviously, in the family dynamics because of the consequences that it generates. Many of the patients with this disease have too many neurological, motor, and psychological symptoms by the time of diagnosis because of the progressive and nonspecific course of the disease, which does not permit the patient or family to notice it early. It is well known that dementia has a particular genetically related factor, which means that people with this disease will transmit it to further generations very probably and also have family history of it in any way. The most important behavior on the recognition and treatment of this disease is prevention. Specially knowing which patients are at risk because of family history of dementia, and also, those who have other diseases that increase the possibilities to develop any type of dementia. “Acting now on dementia prevention, intervention, and care will vastly improve living and dying for individuals with dementia and their families, and in doing so, will transform the future for society” [2]. This is a very important principle that should regulate the acting on this disease, in order to decrease the risks and consequences; and increase the treatment alternatives. Although, by understanding the dementia's course and causes, medicine and science in general are making gigantic steps on this improvement objectives; even if it’s a disease that affects old people; because by caring about old patients, is unavoidable to change younger people’s conscience.Moreover, dementia affects the life of a patient and his or her family significantly. Most of the patients develop neurological, motor, and psychological symptoms by the time of diagnosis that were not noticed earlier because of the slow progression and nonspecific course of the disease. As displayed in Figure 2, visual differences are clearly noted in the patients brain in regards to structure and form. The genetic predisposition of dementia makes it a generational burden, which makes it a familial burden. The ideal mode of recognizing and treating it is based on prevention [7]. The process entails assessing familial history to identify the people at risk of developing the disorder and implementing interventions aimed at limiting its manifestation. Studies show that early dementia prevention and care will improve the quality of life for patients with dementia and their families [7]. This is an important principle that should guide interventions aimed at managing dementia because it will help reduce the risk and effects of the disease as well as increase treatment alternatives. Thus, by understanding the dynamics of dementia (its causes, progression, effects, and treatment options), stakeholders will develop interventions that will improve the quality of life of old patients and change the young people's perceptions of the disease. 

Rheumatoid arthritis

Rheumatoid arthritis is a chronic disorder of the immune system that is characterized by the release of antibodies that attack and destroy body tissues and organs, including the skin, blood vessels, heart, lungs, and muscles [8]. Its most common manifestation is the weakening and inflammation of the joints that gradually progresses to the destruction of the articular cartilage and ankylosis of the joints. It mainly affects the proximal interphalangeal and the metacarpophalangeal (MCP) [8]. The chronic inflammation results in the formation of pannus, which consists of inflammatory cells, granulation tissues, and synovial fibroblasts. The diagnosis of RA is only possible when there are at least four of seven features, which makes early diagnosis difficult because of the variance of signs and symptoms over time. However, early diagnosis and treatment may help to slow down the progression of the disease.

The most reliable form of diagnosing RA currently entails the analysis of the ulnar deviation of MCP joints. The common symptoms of RA include fatigue, depression, morning stiffness that lasts more than one hour, stiffness and/or swelling of the joints such as MCP joints, proximal interphalangeal (PIP) joints, and metatarsophalangeal (MTP) joints [8]. Besides, physical examinations may reveal symptoms such as anemia of chronic disease, weight loss, decreased grip strength, palmar erythema, subcutaneous (rheumatoid) nodules, splenomegaly in cases of Felty syndrome, ulnar deviation of the fingers, swan neck deformity, and boutonniere deformities [8]. 

Additionally, RA is common in middle-aged women. However, it can manifest at any age and is genetically linked to the HLA-DR4 gene. It is also associated with environmental factors and antigens that include smoking, Epstein-Barr virus (EBV), and mycoplasma. As such, people with a family history of RA are at an elevated risk of developing this chronic disease [8]. The study by Le and Bhushan revealed that 80% of those diagnosed with a positive rheumatoid factor have an accumulation of IgM autoantibodies to the Fc portion of autologous IgG [9]. The study also revealed the accumulation of citrullinated proteins (substitution of arginine by citrulline amino acid) that is caused by inflammation and antibodies against citrullinated proteins.

Rheumatoid arthritis occurs when the body's immune system releases antibodies that attack the inner lining of the membranes surrounding the joints known as the synovium [9]. The synovium thickens as a result of the inflammation, which eventually results in the destruction of the cartilage and bone within the joint [9]. An infection often triggers this chain of events in a genetically susceptible person that results in the infiltration of the synovial stroma by CD4+ helper T cells, B cells, plasma cells, dendritic cells, and macrophages [9]. These result in increased vascularity caused by vasodilation and angiogenesis, with superficial hemosiderin deposits and aggregation of organizing fibrin floating in the joint space known as rice bodies. Neutrophils also accumulate in the synovial fluid causing juxta-articular erosions, subchondral cysts, and osteoporosis. 

Overall, these studies show that RA is caused by T- and B-cell responses to an infection in a genetically susceptible person, which results in the formation of pannus and eventual damage of the adjacent cartilage and bone. The Th1 cells release interferon-γ (IFN-γ) to activate macrophages and synovial cells, and the Th17 cells secrete interleukin-17 (IL-17) to recruit monocytes and neutrophils synovial plasma cells produce antibodies against self-antigens such as citrullinated peptides [9]. Cumulatively, these biological reactions and changes result in the development of RA.

Pathogenesis of dementia in rheumatoid arthritis patients

**Figure 3:**
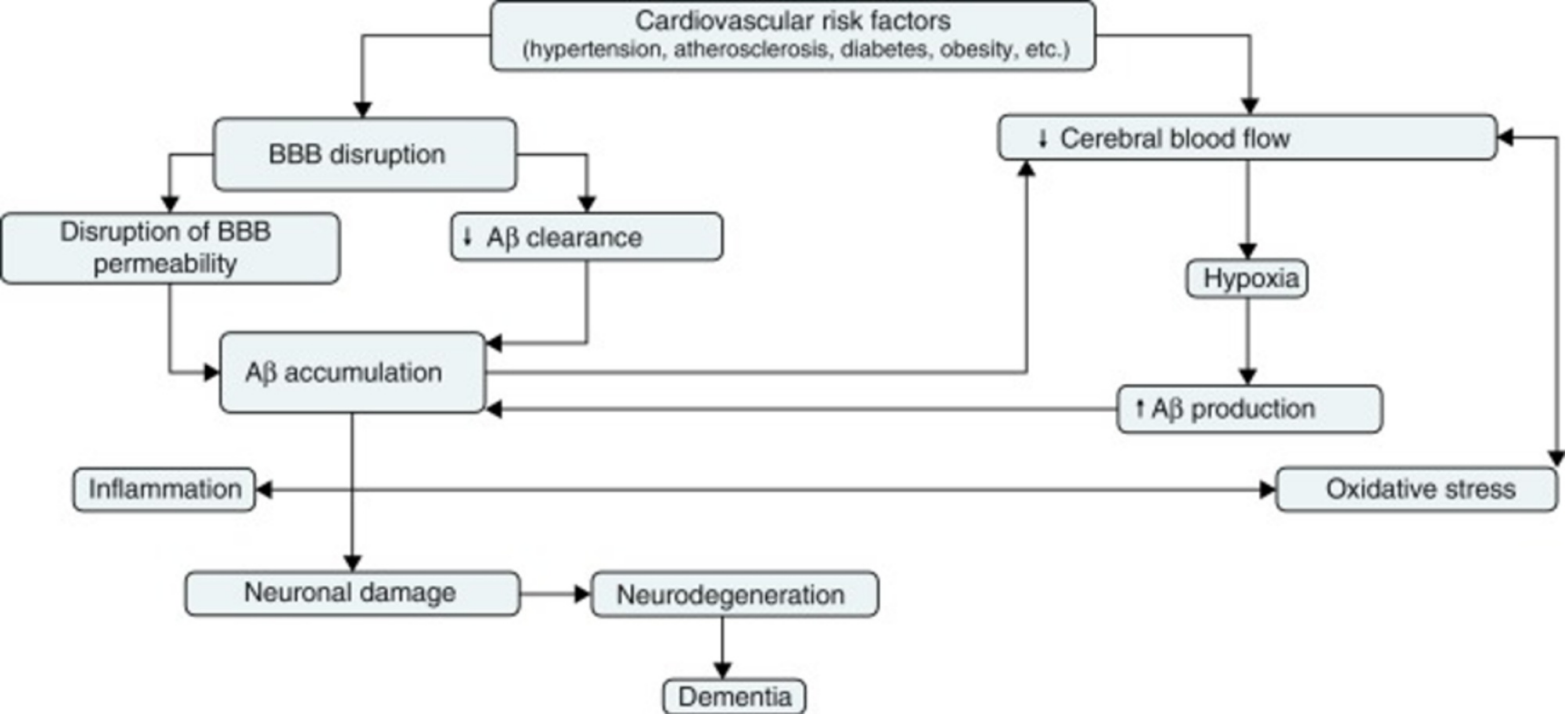
Vascular hypothesis explaining the link between vascular dysfunction and neurodegeneration in AD. AD, Alzheimer's disease. Courtesy: Adapted from Ref. [15].

Patients with RA have displayed symptoms that put them at an elevated risk of developing dementia. A common hypothesis put forward to explain this risk is that the chronic inflammation associated with RA reduces blood circulation. As such, increased inflammation decreases the supply of blood to the brain. Ischemic or hemorrhagic infarcts can cause vascular dementia in many areas of the brain [9-10]. The risk factors for vascular dementia include increased age (over 65 years), hypertension, hyperlipidemia, diabetes, genetics, and smoking/alcohol use. This is shown in Figure 3 as the linkage between vascular dysfunction and neurodegeneration . These risk factors are associated with reduced blood flow that increases the risk of developing dementia. The damage caused by RA leads to atrophy and degeneration of parts of the brain that can affect cognition and behavior [10]. Extensive damage may result in incoherent thought processes, memory loss, and the incapacity to perform routine tasks. A study on the effects of midlife RA revealed an increase in the risk of cognitive impairment and Alzheimer's dementia [10-11]. The main pathological hallmarks of AD are the extracellular deposition of β-amyloid plaques and intra-neuronal neurofibrillary tangles. The increase in the levels of inflammatory signals and cytokines and cerebral inflammation caused by β-amyloid plaque deposition is responsible for the pathophysiology of RA (IL-1, IL-6, and TNF) [12]. The link between AD and the expression of IL-1 reveals a restraint in the function of cholinergic systems and an accumulation of Aβ plaques that initiates dementia and neurofibrillary tangles that are involved in its progression [12-13]. Studies have also shown that medication used by RA patients also increases the risk of developing dementia. Notably, conventional synthetic disease-modifying anti-rheumatic drugs (csDMARDS) such as methotrexate, hydroxychloroquine, and sulfasalazine have a higher risk (1.63-fold) of dementia than those who do not use csDMARDS [13-14]. However, biological therapies, such as tumor necrosis factor (TNF) inhibitors, can lower the risk of dementia [14-15].

 

 

## Conclusions

Overall, this study revealed that the development of RA increases the risk of developing dementia. Firstly, both have a genetic predisposition, which makes their effective management critical to ensuring improved quality of life for both patients and their families. Dementia is associated with the deterioration of one's memory, language processing, visual perception, and other executive bodily functions to the extent that one becomes dependent on a caregiver. RA, on the other hand, is associated with the immune system releasing antibodies that attack healthy body tissues and organs and joints, which leaves one physically impaired. The inflammation affects blood flow in the body, which limits brain functions because of limited oxygen supply. Other vital organs also lack adequate blood flow, which limits their functioning. These changes increase the risk of developing dementia. The study also revealed that these health issues manifest mostly in older people aged above 65. The findings reinforce the need to develop diagnostic procedures that will enable early diagnosis and commencement of treatment to slow down the progression of both disorders. Furthermore, there is a need for increased awareness about the causality and risk factors of both diseases, especially among young people and at-risk populations to promote lifestyle change and increased uptake of primary care services. These will help reduce the prevalence and progression of both RA and dementia. 
